# Identification of IGF-1 Effects on White Adipose Tissue and Hippocampus in Alzheimer’s Disease Mice via Transcriptomic and Cellular Analysis

**DOI:** 10.3390/ijms25052567

**Published:** 2024-02-22

**Authors:** Young-Kook Kim, Danbi Jo, Archana Arjunan, Yeongseo Ryu, Yeong-Hwan Lim, Seo Yoon Choi, Hee Kyung Kim, Juhyun Song

**Affiliations:** 1 Department of Biochemistry, Chonnam National University Medical School, Hwasun 58128, Jeollanamdo, Republic of Korea; ykk@jnu.ac.kr (Y.-K.K.); ryu0seoo@gmail.com (Y.R.); yeonghwan2609@gmail.com (Y.-H.L.); 2 Biomedical Science Graduate Program (BMSGP), Chonnam National University, Hwasun 58128, Jeollanamdo, Republic of Korea; danbijo0818@gmail.com (D.J.); sy20180433@gmail.com (S.Y.C.); 3 Department of Anatomy, Chonnam National University Medical School, Hwasun 58128, Jeollanamdo, Republic of Korea; archanaibms@gmail.com; 4 Department of Endocrinology and Metabolism, Department of Internal Medicine, Chonnam National University Medical School, Hwasun 58128, Jeollanamdo, Republic of Korea; albeppy@gmail.com

**Keywords:** Alzheimer’s disease (AD), insulin-like growth factor-1 (IGF-1), RNA sequencing, hippocampus, adipose tissue

## Abstract

Alzheimer’s disease (AD) stands as the most prevalent neurodegenerative disorder, characterized by a multitude of pathological manifestations, prominently marked by the aggregation of amyloid beta. Recent investigations have revealed a compelling association between excessive adiposity and glial activation, further correlating with cognitive impairments. Additionally, alterations in levels of insulin-like growth factor 1 (IGF-1) have been reported in individuals with metabolic conditions accompanied by memory dysfunction. Hence, our research endeavors to comprehensively explore the impact of IGF-1 on the hippocampus and adipose tissue in the context of Alzheimer’s disease. To address this, we have conducted an in-depth analysis utilizing APP/PS2 transgenic mice, recognized as a well-established mouse model for Alzheimer’s disease. Upon administering IGF-1 injections to the APP/PS2 mice, we observed notable alterations in their behavioral patterns, prompting us to undertake a comprehensive transcriptomic analysis of both the hippocampal and adipose tissues. Our data unveiled significant modifications in the functional profiles of these tissues. Specifically, in the hippocampus, we identified changes associated with synaptic activity and neuroinflammation. Concurrently, the adipose tissue displayed shifts in processes related to fat browning and cell death signaling. In addition to these findings, our analysis enabled the identification of a collection of long non-coding RNAs and circular RNAs that exhibited significant changes in expression subsequent to the administration of IGF-1 injections. Furthermore, we endeavored to predict the potential roles of these identified RNA molecules within the context of our study. In summary, our study offers valuable transcriptome data for hippocampal and adipose tissues within an Alzheimer’s disease model and posits a significant role for IGF-1 within both the hippocampus and adipose tissue.

## 1. Introduction

Alzheimer’s disease (AD) is the most common neurodegenerative disorder worldwide, accounting for over 70% of dementia cases, with approximately 55 million people diagnosed worldwide as of 2019 [1]. AD is commonly characterized by memory loss, excessive amyloid beta (Aβ) peptide accumulation, hyperphosphorylated tau proteins, microglia activation, and neuronal cell death [2,3,4]. Recently, the relationship between metabolic diseases and AD neuropathology has been highlighted, and studies on metabolic imbalance-related mechanisms to treat AD neuropathological symptoms are emerging [5,6,7].

Obesity is one of the metabolic diseases and is accompanied by an excessive accumulation of adipose tissue, increased adipocytokine and inflammatory cytokines in the serum [8], impaired neurovascular systems, and hippocampal neuronal dysfunction [9], leading to cognitive impairment [10]. In this respect, recently researchers have tried to study adipose and brain tissues in AD [11,12].

Adipose tissue contains many hormones, triglycerides, adipocytes, and macrophages [13]. Specifically, visceral white adipose tissue contributes to reduced gray matter volume [14], dysregulated lipid metabolism [15], and cerebral vascular dysfunction [16], leading to the onset of AD [17]. One epidemiological study suggested that the high accumulation of visceral adipose tissue leads to the risk for AD [18]. Current studies have demonstrated that several molecules secreted from adipose tissue, such as adiponectin and leptin, are strongly related to the progression of AD [11,19]. Although further studies on the relationship between adipose tissue and brain tissue in AD are needed in order to solve the neurological issues caused by metabolic imbalance, analyses of changed genes and specific mechanisms in the two tissues were not fully understood until now.

Among the changes in metabolic signaling under a metabolic imbalance condition, low systemic insulin sensitivity and brain insulin resistance lead to neurological issues such as hippocampal memory dysfunction [20,21] and depressive mood problems [22,23]. Insulin-like growth factor 1 (IGF-1) is an endocrine growth polypeptide hormone that shares about 50% similarity with the proinsulin structure [24]. IGF-1 is released from the liver, muscle, heart, pancreatic beta cells, brain, and adipose tissues [25]. Several studies have demonstrated that IGF-1 regulates the glucose metabolism, skeletal muscle metabolism, atheroprotective responses, and neuroprotective signaling [26,27,28]. Previous studies have reported that reduced levels of IGF-1 in the blood of patients with metabolic disorders are a common feature [29,30]. Furthermore, several studies have demonstrated that dysregulation and low sensitivity to insulin or IGF-1 increase the onset of AD and aggravate severe AD neuropathology [31,32,33].

Recently, as the incidence of dementia accompanying metabolic disorders increases, the interest in the effects of IGF-1, which exhibits various ameliorating effects in metabolic disorders, on the central nervous system (CNS) is increasing [34,35,36]. In the CNS, insulin-like growth factor-I receptors (IGF-IRs) are widely expressed in various brain regions, and systemic IGF-1 can cross the blood–brain barrier and enter the brain [37,38]. Some studies have shown that IGF-1 increases hippocampal neurogenesis, memory functions, synaptogenesis, spine maturation, and neurite growth [39], and reduces the amyloid beta (Aβ) clearance capacity [40] and hippocampal volume [41], all of which are involved in neuropathology and psychiatric diseases [42,43].

Some studies have suggested that IGF-1 enhances neurogenesis in the hippocampus [44] and increases neurovascular structure stability [45], brain atrophy [46], spatial learning, and memory ability [47,48] in the CNS. Also, it has been reported that IGF-1 can reduce tau hyperphosphorylation [49] and Aβ clearance [50] and promote the cell survival related to insulin signaling by IGF-1R [51]. Since the brain and visceral white adipose tissues are associated with the regulation of insulin resistance [52], both the brain and adipose tissues should be investigated to understand metabolic neurological diseases.

In this study, we first confirmed the change in behavior of APP/PS2 mice, an AD transgenic mice model [53], after low-dose IGF-1 injections. Subsequently, we examined genetic alterations in the hippocampal and visceral adipose tissues. We assessed whether the fat browning- and mitochondria-related functions in the adipose tissue and neuronal damage and neuroinflammatory response in the hippocampus of APP/PS2 mice were influenced by the IGF-1 injections. Taken together, we expect that our data will help to understand hippocampal and adipose tissue dysfunctions in AD.

## 2. Results

**Figure 1:**
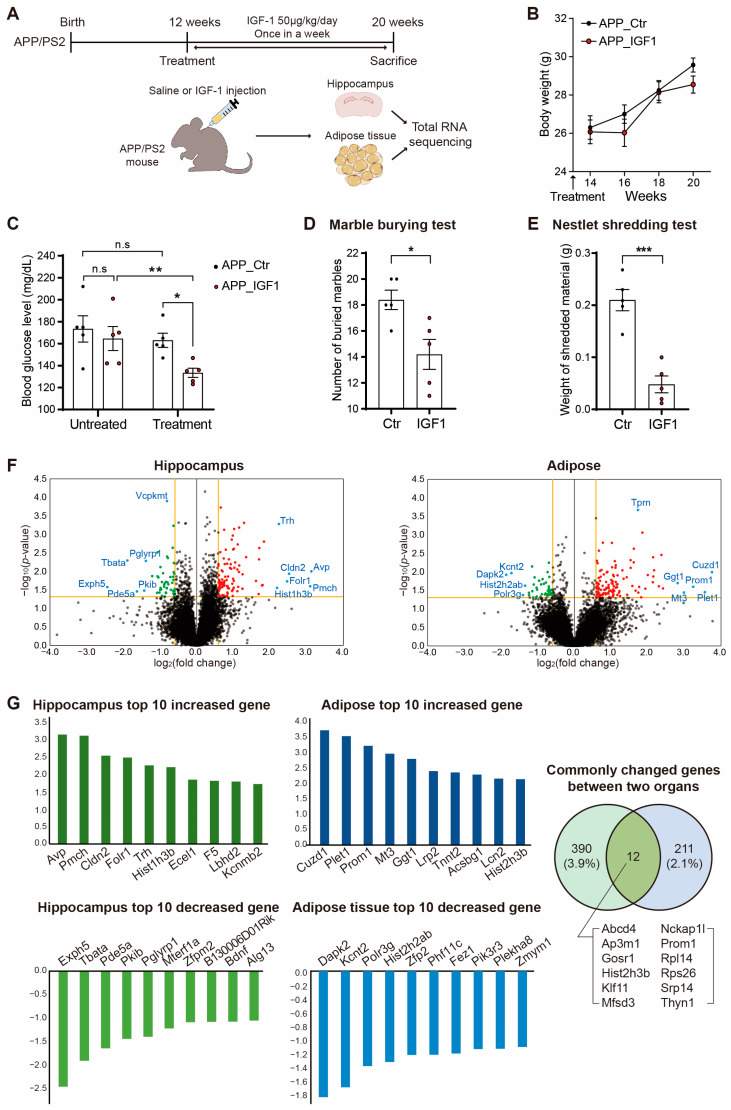
Transcriptome analysis of the hippocampus and adipose tissues from IGF-1-injected APP/PS2 mice. (**A**) Schematic figure of the study design. (**B**) Body weight changes after the injection of IGF-1. (**C**) The blood glucose levels in the serum of IGF-1-injected APP/PS2 mice. (**D**) The marble-burying behavior test and (**E**) nestle shredding test of IGF-1-injected APP/PS2 mice. We used five mice in each group (n = 5) for the experiments in (**B**–**E**). (**F**) Volcano plots of the hippocampus and adipose tissue in IGF-1-injected APP/PS2 mice. The *X*-axis represents the log_2_-transformed fold change of each group, and the *Y*-axis represents the −log_10_(*p*-value) value. The colored dots depict the significantly changed genes. The vertical yellow bar indicates the −log_2_(fold change) of 0.6, while the horizontal yellow bar indicates the −log_10_(*p*-value) of 1.3, respectively. (**G**) The top ten increased and decreased genes among those with significant expression changes in the hippocampus and adipose tissue of IGF-1-injected APP/PS2 mice. An unpaired two-tailed *t*-test with Welch’s correction was used for the statistical analysis. n.s, not significant, * *p* < 0.05, ** *p* < 0.01, *** *p* < 0.005. The Ctr group indicates the APP/PS2 control mice group with saline injection, and the IGF-1 group indicates the IGF-1-injected APP/PS2 mice group.

We injected three-month-old APP/PS2 mice with IGF-1 subcutaneously at a dose of 50 μg/kg/day once weekly for two months, similar to the previous study [54] (Figure 1A). The APP/PS2 mice with IGF-1 injections had lower body weights and blood glucose levels than the normal saline-injected APP/PS2 control mice (Figure 1B,C). We conducted two behavior tests, the marble burying test and the nestlet shredding test, to confirm behavioral alterations between the two groups before being sacrificed (Figure 1D,E). The number of buried marbles for the APP/PS2 mice with IGF-1 injections was significantly lower than that for the APP/PS2 control mice (Figure 1D). The nestlet shredding test showed that the weights of the shredded material were lower for the APP/PS2 mice with IGF-1 injections than for the APP/PS2 control mice (Figure 1E). All the mice were sacrificed, and the hippocampus and visceral adipose tissue were obtained for subsequent experiments (Figure 1A).

We performed an RNA sequencing analysis using the RNA extracted from the hippocampal and adipose tissues. The gene expression changes were visualized as a volcano plot (Figure 1F). We also indicated several genes with high expression levels and statistically significant changes in each tissue. As depicted in the graph, the expressions of genes including the thyrotropin-releasing hormone (*Trh*), arginine vasopressin (*Avp*), and pro-melanin-concentrating hormone (*Pmch*) were significantly changed in the hippocampus, whereas the expression of genes such as taperin (*Tprn*), CUB and zona pellucida like domains 1 (*Cuzd1*), prominin 1 (*Prom1*), and placenta expressed transcript 1 (*Plet1)* were significantly changed in the mouse adipose (Figure 1F).

Among the genes with a *p*-value less than 0.05, we selected the top ten increased genes and the top ten decreased genes in the hippocampal and adipose tissues based on fold change (Figure 1G). The expression of *Avp*, *Pmch*, *Cldn2*, *Folr1*, *Trh*, *Hist1h3b*, *Ecel1*, *F5*, *Lbhd2*, and *Kcnmb2* were significantly increased in the hippocampus, whereas the expression of *Exph5*, *Tbata*, *Pde5a*, *Pkib*, *Pglyrp1*, *Mterf1a*, *Zfpm2*, *B130006D01Rik*, *Bdnf*, and *Alg13* were significantly decreased in the hippocampus (Figure 1G). The expression of *Cuzd1*, *Plet1*, *Prom1*, *Mt3*, *Ggt1*, *Lrp2*, *Tnnt2*, *Acsbg1*, *Lcn2*, *and Hist2h3b* were significantly increased in the adipose tissue, whereas the expression of *Dapk2*, *Kcnt2*, *Polr3g*, *Hist2h2ab*, *Zfp2*, *Phf11c*, *Fez1*, *Pik3r3*, *Plekha8*, and *Zmym1* were significantly decreased in the adipose tissue (Figure 1G). Moreover, the genes including *Abcd4*, *Ap3m1*, *Gosr1*, *Hist2h3b*, *Klf11*, *Mfsd3*, *Nckap1l*, *Prom1*, *Rpl14*, *Rps26*, *Srp14*, and *Thyn1* showed a common expression change in both the hippocampus and adipose tissues.

**Figure 2:**
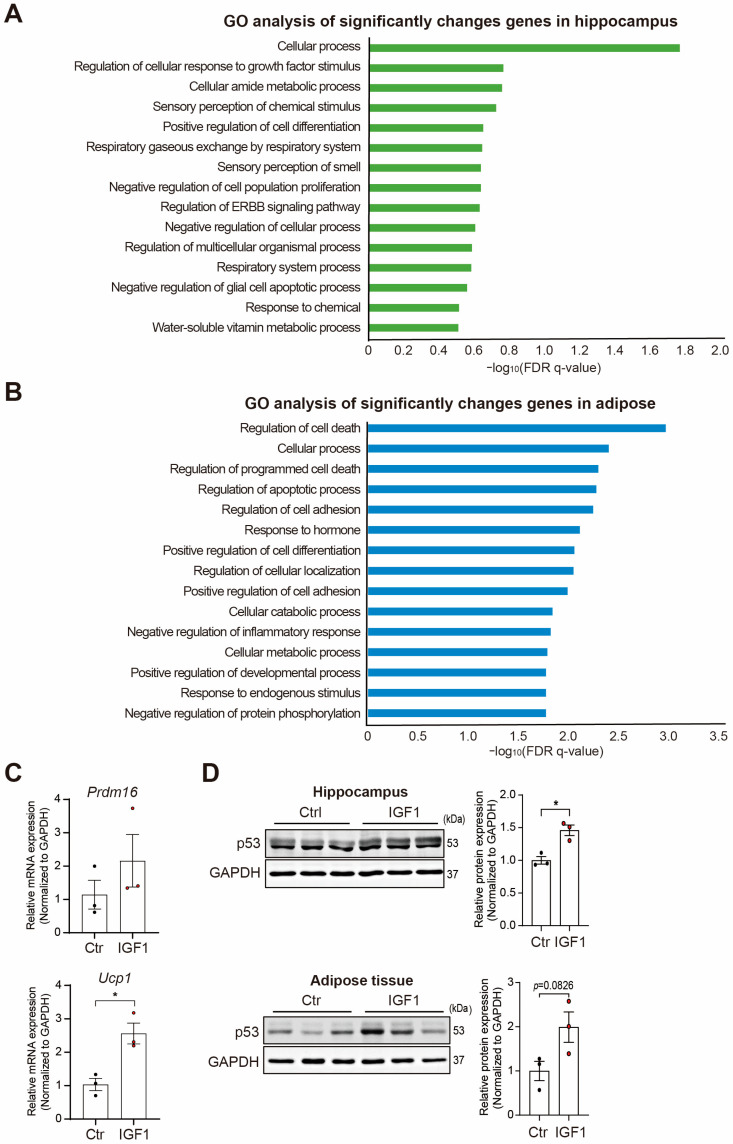
Functional analysis and expression measurements of the significantly changed genes in the hippocampus and adipose tissue. (**A**) The GO analysis for the significantly changed genes in the hippocampus. The top 15 GO terms based on the false discovery rate (FDR) q-values are shown. (**B**) The GO analysis for the significantly changed genes in the adipose tissue. The top 15 GO terms based on the FDR q-values are shown. (**C**) Measurements of the mRNA levels of fat browning-related genes, including *Prdm16* and *Ucp1*, in the adipose tissue of IGF-1-injected APP/PS2 mice (n = 3). (**D**) Western blot analysis of p53 in the hippocampus and adipose tissue of APP/PS2 mice injected with IGF-1. All data are represented as the mean ± S.E.M (n = 3). An unpaired two-tailed *t*-test with Welch’s correction was used for the statistical analysis. * *p* < 0.05. The Ctr group indicates the APP/PS2 control mice group, and the IGF-1 group indicates the IGF-1-injected APP/PS2 mice group. Full-length blots are presented in Supplementary Figure S2.

Next, we performed a GO analysis to identify the molecular and cellular pathways related to the genes that were significantly changed in the hippocampus and adipose tissue (Figure 2A,B). From the analysis of the hippocampal data, there were 489 protein-coding genes with a significant change in their expression and a *p*-value less than 0.05. From the analysis of the adipose tissue, there were 239 protein-coding genes with a significant change in their expression and a *p*-value less than 0.05. Among these genes, 200 were selected in the order of the smallest *p*-value for the GO analysis. The GO analysis of the hippocampal genes revealed several enriched terms, and the term cellular process was the most significantly enriched one. For the adipose genes, the terms related to cell death were prominently enriched.

Since the signaling related to the cellular catabolic process and metabolic process was enriched in the adipose tissue, we measured the mRNA levels of fat browning-related genes such as *Prdm16* and *Ucp1* in the adipose tissue of the control and IGF-1-injected groups (Figure 2C). As expected, in the IGF-1 injected APP/PS2 mice, the mRNA levels of fat browning-related genes were increased compared with the control mice.

The cell death- and apoptosis-related terms were enriched in both tissues. Accordingly, we measured the level of the p53 protein, which is a key factor in these pathways. Our result shows that the protein level of p53 was significantly increased in the hippocampus and adipose tissue by the IGF-1 injections (Figure 2D). Also, we determined the change of cytokines and adipokines in the plasma of IGF-1-treated APP/PS2 mice using each array kit (Supplementary Figure S1). The level of C5/C5a was slightly decreased and the level of M-CSF and TIMP-1 was slightly increased in the plasma of the IGF-1-treated APP/PS2 mice. Among the adipokines, the levels of ICAM-1, RAGE, and Serpin E1 were a little increased and the level of IGF-1, IGFBP-3, and TIMP-1 was slightly reduced in the plasma of the IGF-1-treated APP/PS2 mice (Supplementary Figure S1).

**Figure 3:**
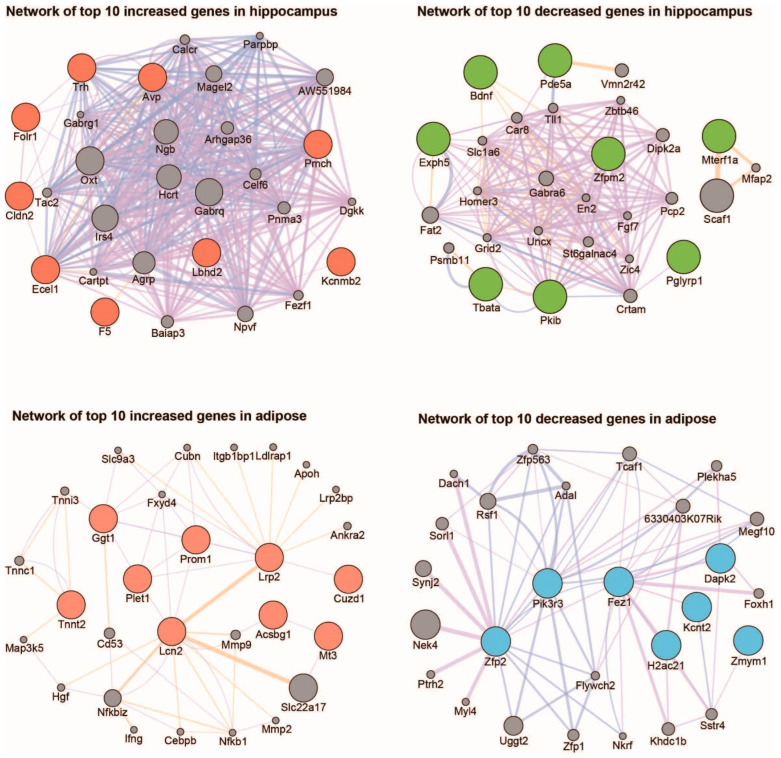
Analysis of the intracellular interaction networks for the protein-coding genes whose expressions were significantly changed in the hippocampus and adipose tissue after IGF-1 treatments. Interaction networks were drawn for the top ten most significantly increased and decreased genes in the hippocampus and adipose tissue.

To check the interaction network formed between the protein-coding genes whose expressions were significantly changed by the IGF-1 injections in each tissue, a network analysis was performed on the selected genes (Figure 3). We analyzed the interactions between the protein-coding genes using GeneMANIA and visualized them using Cytoscape [55,56]. By analyzing the top ten genes that increased or decreased the most significantly in each group, it was possible to predict what kind of interaction network these genes formed in the hippocampus and adipose (Figure 3). Interestingly, these gene groups showed different network patterns in the hippocampus and adipose from each other. In the hippocampus, the degree of interaction between each gene was less than that of the adipose genes, and fewer genes formed a hub on the network. In contrast, genes that were significantly changed in the adipose tissue showed a network that had more interactions with each other, and the number of genes occupying a hub was higher than that of the hippocampus genes (Figure 3).

**Figure 4:**
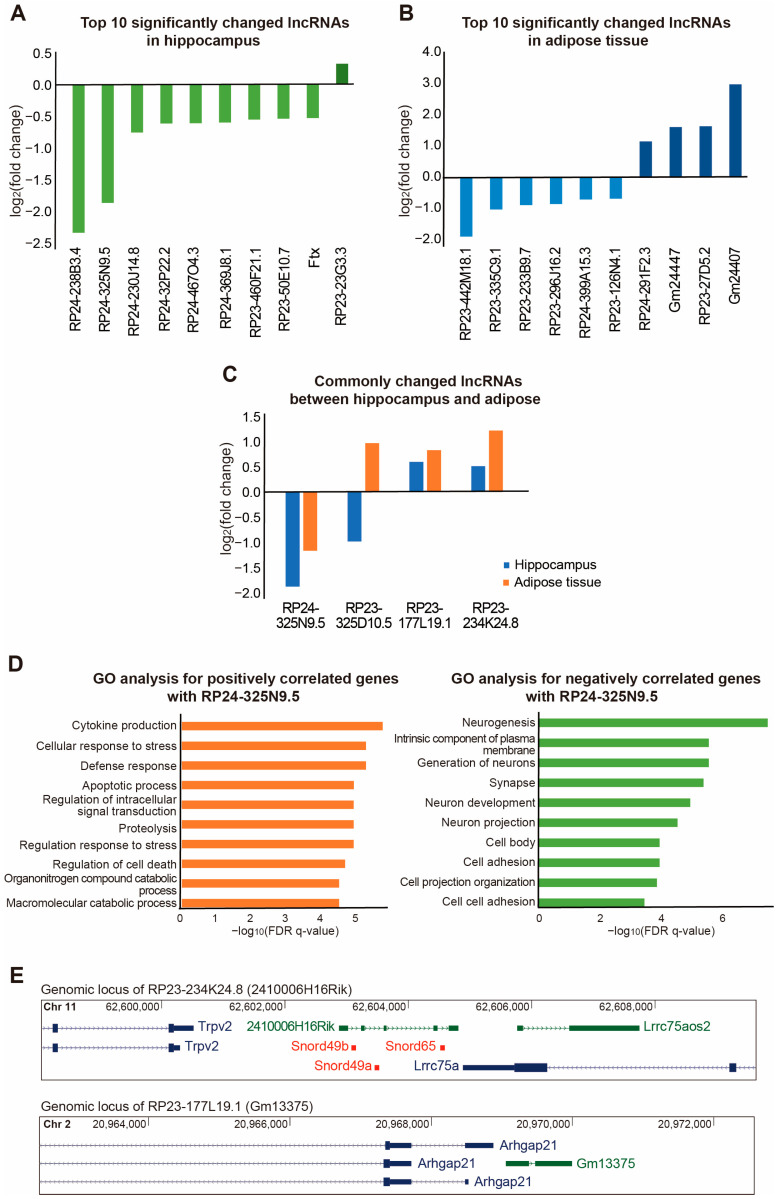
Expression changes of lncRNAs and their functional analysis in the tissue samples of IGF-1-treated APP/PS2 mice. (**A**) The graph shows the top ten lncRNAs whose expressions were most significantly changed in the hippocampus. (**B**) The graph shows the top ten lncRNAs whose expressions were most significantly changed in the adipose tissue. (**C**) The expression changes of four lncRNAs with a significant (*p* < 0.1) change in both the hippocampus and adipose tissue. (**D**) To predict the intracellular function of the lncRNA RP24-325N9.5, protein-coding genes with a positive correlation with this lncRNA (left) and those with a negative correlation with this lncRNA (right) were selected. A GO analysis was performed on the selected genes using MSigDB. (**E**) The genomic information near the DNA locus of the RP23-234K24.8 and RP23-177L19.1 lncRNA genes was obtained from the UCSC Genome Browser (http://genome.ucsc.edu, accessed on 15 November 2022). The blue color indicates protein-coding genes, the green color indicates lncRNA genes, and the red color indicates small non-coding RNA genes.

We also identified significantly changed long non-coding RNAs (lncRNAs) in the hippocampus (Figure 4A) and adipose tissue (Figure 4B). lncRNAs are non-coding RNAs with a length longer than 200 nucleotides and they regulate diverse cellular mechanisms. For the analysis of lncRNA expression, we first selected all the genes except for the protein-coding genes from the RNA-seq results based on the gene class presented in the annotation of the GENCODE. The most significantly changed genes in the hippocampus and adipose tissue were selected in the order of the *p*-value of the change in expression level (Figure 4A,B). Our data revealed that the top ten significantly changed lncRNAs in the hippocampus were RP24-238B3.4, RP24-325N9.5, RP24-230J14.8, RP23-32P22.2, RP24-467O4.3, RP24-369J8.1, RP23-460F21.1, RP23-50E10.7, Ftx, and RP23-23G3.3 (Figure 4A). The top ten significantly changed lncRNAs in the adipose tissue were RP23-442M18.1, RP23-335C9.1, RP23-233B9.7, RP23-296J16.2, RP24-399A15.3, RP23-126N4.1, RP24-291F2.3, Gm24447, RP23-27D5.2, and Gm24407 (Figure 4B).

Moreover, we selected the most significantly and commonly changed lncRNAs in both the hippocampus and adipose tissue (Figure 4C). Based on the criteria, we found four candidate lncRNAs: RP24-325N9.5, RP23-325D10.5, RP23-177L19.1, and RP23-234K24.8 (Figure 4C). The expression of RP24-325N9.5 was commonly reduced in the hippocampus and adipose tissue, while the expressions of RP23-177L19.1 and RP23-234K24.8 were commonly increased in the hippocampus and adipose tissue (Figure 4C).

Among the lncRNAs, RP24-325N9.5 was the most significantly changed lncRNA and was selected, and the function was predicted. For this purpose, the protein genes that had a high correlation with RP24-325N9.5 based on their expression levels in the tissue samples were selected. As a result of the analysis, the GO terms related to cytokine production, the cellular response to stress, defense response, apoptotic process, regulation of intracellular signal transduction, proteolysis, the regulation response to stress, regulation of cell death, organonitrogen compound catabolic process, and the macromolecular catabolic process were enriched in the protein genes whose expressions were changed in the same direction as RP24-325N9.5 in the hippocampus and adipose tissue (Figure 4D). In contrast, the protein-coding genes whose expressions changed in the opposite direction of RP24-325N9.5 were enriched with GO terms related to neurogenesis, the intrinsic component of the plasma membrane, the generation of neurons, synapse, neuron development, neuron projection, cell body, cell adhesion, cell projection organization, and cell–cell adhesion (Figure 4D). Therefore, this lncRNA is likely to act positively on the pathways related to inflammation or immune response, and it can be inferred that the pathways related to neurogenesis could be negatively regulated by this lncRNA.

Interestingly, the commonly increased lncRNAs, including RP23-234K28.8 and RP23-177L19.1, resided in close proximity to other protein-coding or non-coding RNA genes at their genomic locus (Figure 4E). Since many lncRNAs are known to regulate nearby genes at the transcription level, it is plausible that these lncRNAs may have a similar mechanism in the hippocampus and adipose tissue.

**Figure 5:**
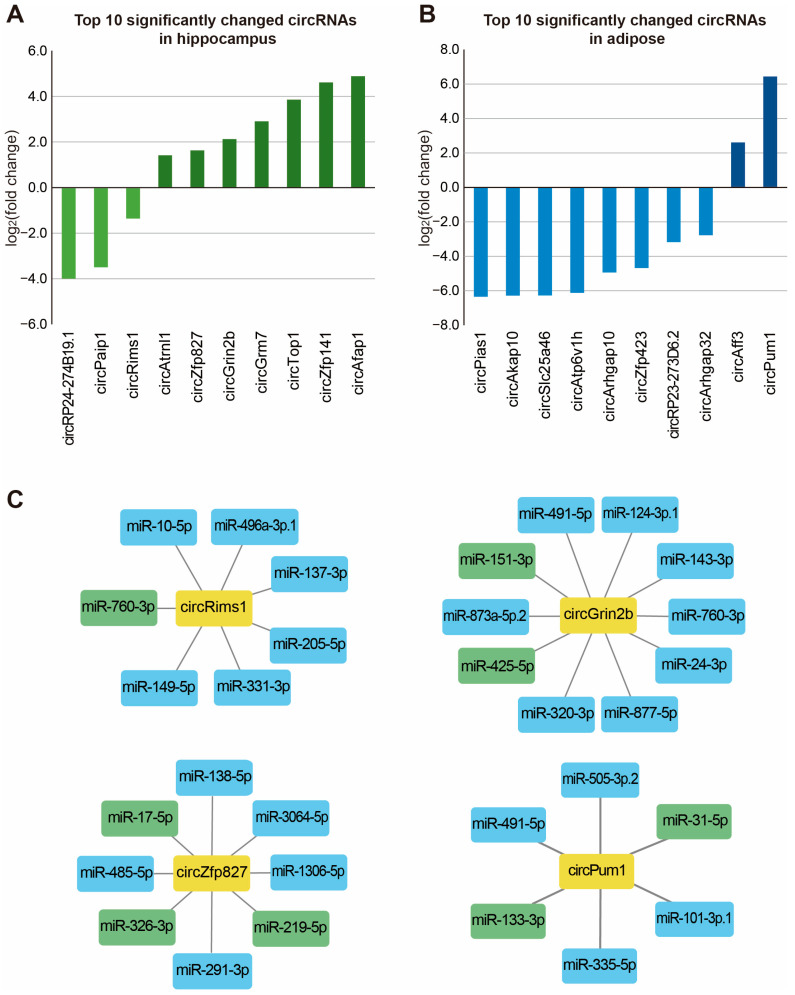
Expression changes of circRNAs and their functional analysis in the tissue samples of IGF-1-treated APP/PS2 mice. (**A**) The graph shows the top ten circRNAs whose expressions were most significantly changed in the hippocampus. (**B**) The graph shows the top ten circRNAs whose expressions were most significantly changed in the adipose tissue. (**C**) Among the circRNAs whose expressions were significantly changed in the hippocampus or adipose tissue by the IGF-1 treatments, the regulatory relationship with miRNAs was predicted for the circRNAs whose function in each tissue had been previously studied. The miRNAs shown in the green box indicate the presence of an ‘8mer’ site in the circRNA sequence, and miRNAs shown in the blue box indicate the presence of a ‘7mer-m8’ site.

Since circular RNAs (circRNAs) are likely to be involved in various intracellular signaling pathways, we also calculated the change in circRNA expression by the IGF-1 treatments in the hippocampus and adipose tissue (Figure 5). We found that the expression levels of various circRNAs were changed in each tissue by the IGF-1 injections. We selected the ten circRNAs whose expressions were most significantly changed by the IGF-1 injections in the hippocampus and adipose tissue based on their *p*-values (Figure 5A,B). To identify the circRNAs whose expressions were commonly changed in the hippocampus and adipose tissue, we selected circRNAs with a *p*-value of 0.1 or less from the hippocampus and adipose tissue. Interestingly, there were no circRNAs with a significantly altered expression in the hippocampus and adipose tissue. Therefore, it can be expected that the change in circRNA expression is more tissue-specific than other gene classes.

We investigated previous reports to predict the functional roles of the selected circRNAs in the hippocampus and adipose tissue. For the candidate circRNAs selected from the adipose tissue, circRNAs that were functionally studied in the adipose tissue or that regulate functions related to the role of adipose tissue were selected. For the candidate circRNAs selected from the hippocampus, circRNAs that were studied in brain cells or whose functions were reported to be related to neurological diseases were selected. As a result of the literature analysis, circPum1 was selected in the adipose tissue, and circRims1, circZfp827, and circGrin2b were selected in the hippocampus. It has been reported that circPUM1 is highly expressed in insulin-treated granulosa cells, and it promotes polycystic ovary syndrome progression by sponging miR-760 [57]. The downregulation of circRims1 has been observed in vascular dementia rats [58], while circZNF827 negatively regulates neuronal differentiation [59]. Finally, circGrin2b has been identified to be upregulated in the dorsolateral prefrontal cortex of patients with a cocaine use disorder [60].

To predict how these circRNAs function in cells, we analyzed the miRNAs to which these circRNAs are predicted to bind. Most circRNAs are distributed in the cytoplasm, and it is known from previous studies that cytoplasmic circRNAs act mainly by inhibiting miRNAs [61]. Therefore, we used the TargetScan algorithms to analyze which miRNA binding sites exist in the selected circRNAs [62]. It was found that the four selected circRNAs had binding sites on various miRNAs (Figure 5C). Thus, future studies need to analyze the regulatory relationship between these circRNAs and the miRNAs under their influence in the context of IGF-1 injections.

**Figure 6:**
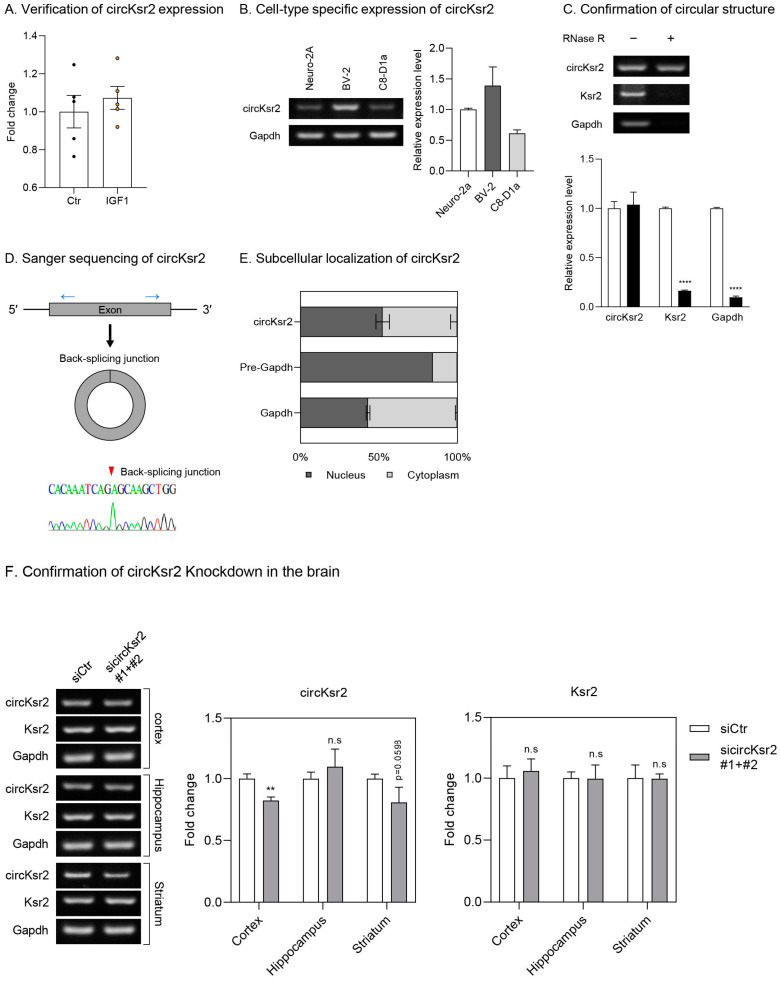
Verification of circKsr2 expression in CNS cells and protein levels in an IGF-1-injected APP mouse brain hippocampus. (**A**) Verification of circKsr2 expression changes in the hippocampus of IGF-1-injected APP mice (n = 5). Expression of circKsr2 was normalized by that of *Gapdh*. Data are displayed as mean ± SEM. (**B**) Measurement of circKsr2 in three different brain cell lines of the mouse. The expression levels are presented as the relative expression compared to the Neuro-2a cell line (n = 3). Data are displayed as mean ± SEM (**C**) Confirmation of circular structure of circKsr2 in Neuro-2A. After RNase R treatment, circKsr2 was measured by semiquantitative PCR using divergent primers for circKsr2 (n = 3). The circRNA was not affected by RNase R, but the linear RNAs including *Ksr2* and *Gapdh*, were degraded. Data are displayed as mean ± SEM. Student’s test was used for statistical analysis. **** *p* < 0.0001. (**D**) The illustration showing the positions of PCR primers for circRNA amplification. The red arrow indicates the back-splicing junction of circKsr2 confirmed by Sanger sequencing. (**E**) Subcellular localization of circKsr2 in Neuro-2A. The data are illustrated as a percentage of relative expression between the nucleus and cytoplasm. Pre-*Gapdh* was used as a control of nuclear RNA, and *Gapdh* was used as a control of cytoplasmic RNA. Data are displayed as mean ± SEM. (**F**) Changes in circKsr2 and *Ksr2* expression following circKsr2 silencing in the cortex, hippocampus, and striatum of APP mice injected with IGF-1. The expression of circKsr2 and *Ksr2* was normalized by that of *Gapdh*. Data are displayed as mean ± SEM. Student’s test was used for statistical analysis. ** *p* < 0.01, n.s: not significant.

To study the function of novel circRNAs in IGF-1-injected APP mice, we selected circRNAs with significantly altered expression in the hippocampus that had not been previously studied. In this process, we selected circKsr2 and found that the expression of this circRNA was significantly increased in the hippocampus of IGF-1-injected APP mice (Figure 6A). When we checked its expression in three different brain cell types, circKsr2 was relatively highly expressed in the BV-2 microglial cell type (Figure 6B). RNase R treatment experiments confirmed the circular structure of circKsr2 (Figure 6C), and sequencing validated the back-splicing junction (Figure 6D). Subcellular localization confirmed that circKsr2 was mainly distributed in the cytoplasm (Figure 6E). To analyze the function of circKsr2, we injected siRNA against circKsr2 into the brains of mice using an osmotic pump. As a result, the expression of circKsr2 was suppressed in the cortex and striatum, while the amount of the host gene, Ksr2 mRNA, remained unchanged (Figure 6F).

**Figure 7:**
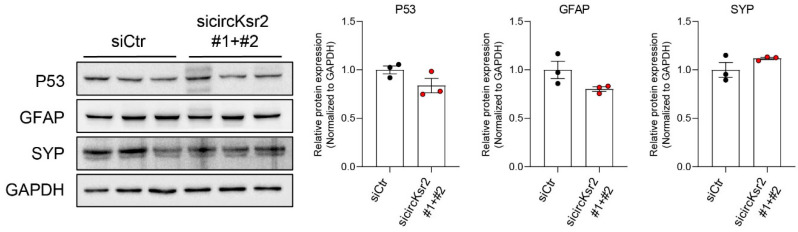
Measurement of protein levels in siRNA circKsr2 knockout mouse hippocampus. Western blot analysis of proteins in the APP mice hippocampus following circKsr2 knockdown in the mouse hippocampus. P53, GFAP, and SYP protein expression. All data are represented as mean ± S.E.M (n = 3). An unpaired two-tailed *t*-test with Welch’s correction was used for the statistical analysis. The siCtr group indicates the siRNA control mice group, and the sicircKsr2 #1+#2 group indicates the circKsr2 siRNA mice group. Full-length blots are presented in Supplementary Figure S4.

Furthermore, we conducted an analysis of protein expression changes, specifically assessing the levels of p53, GFAP, and SYP within the hippocampus of APP mice with suppressed circKsr2 expression. As depicted in Figure 2D, our findings indicate a significant increase in p53 levels in the hippocampus following IGF-1 treatment. Notably, when circKsr2 expression is suppressed, a discernible decrease in p53 levels is observed (Figure 7). Moreover, we observed alterations in the protein expression of the glial fibrillary acidic protein (GFAP), a prominent component of intermediate filaments and a specific marker for astrocytes. Its expression was found to be modestly diminished. In contrast, the expression of synaptophysin (SYP), a presynaptic marker protein, exhibited a slight increase upon the inhibition of circKsr2 (Figure 7). These findings collectively suggest that circKsr2 may play a significant regulatory role within the hippocampus. Full-length blots are presented in Supplementary Figure S3.

## 3. Discussion

In this study, we performed a transcriptome analysis on hippocampal and adipose tissues after low-dose IGF-1 injections for two months in APP/PS2 mice to understand the role of IGF-1 in AD (Supplementary Figure S5). In the APP/PS2 mice, we observed slightly lower blood glucose levels and less weight gain after two months of IGF-1 injections compared with the normal saline-injected APP/PS2 control mice (Figure 1). This data indicated that IGF-1 affects the stable glucose metabolism and maintenance of weight gain in AD, suggesting that AD patients and AD model mice exhibit a glucose tolerance, impaired insulin sensitivity [63], and a poor systemic metabolism [64,65]. Recent studies have suggested that maintaining a proper weight and stable glucose level can help prevent the progression of AD [65,66]. We conducted two types of behavior tests: the marble burying test, known as a test for anxiety, obsessive–compulsive behavior, and repetitive behaviors [67,68], and the nestlet shredding test, known as a test for motivation, psychiatric disorders [69,70], and dopamine-related depressive behaviors [71] (Figure 1D,E). Our data showed that the administration of IGF-1 improved both behavioral assessment scores in the APP/PS2 mice (Figure 1D,E). Based on these data, we hypothesize that IGF-1 influences some neuropsychiatric alterations and mood-behavioral changes in AD.

Several studies have shown that IGF-1 impacts the anxiety response, obsessive behavior, and depressive mood disorder [72,73]. Some neuropsychiatric symptoms, such as obsessive–compulsive disorder and depressive symptoms, are related to the onset and progression of AD and mild cognitive disorders [74,75]. One study has reported that obsessive–compulsive behavior is an early physical sign of AD and can be observed in APP/PS1 mice aged three to five months [76]. Other studies have shown that anxiety and depressive behaviors share neural circuits with common neuropathological diseases with cognitive impairment [77,78,79]. Based on numerous studies, psychiatric emotional regulation contributes to various pathways such as neuronal cell death, synaptic plasticity, axon branching, neuronal differentiation, the inflammatory response, glial cell death, and ultimately cognitive abilities [80,81], leading to the onset of neurodegenerative diseases [82]. Given our behavior tests, we assume that IGF-1 may modulate neuropsychiatric and neuropathologic pathways in AD.

We investigated the altered transcriptome in the hippocampus and adipose tissue of the IGF-1-injected APP/PS2 mice through an RNA sequencing analysis. We analyzed genes with significant changes in the hippocampus and adipose tissue (Figure 1F). We found that some genes, such as *Trh*, *Avp*, *and Pmch* were differentially expressed in the IGF-1 injected APP/PS2 mice hippocampi (Figure 1F). TRH is related to the increased secretion of γ-Aminobutyric acid sub-type A (GABA). GABA, in the hippocampus, regulates cognition, mood, and sleep patterns [83]. Also, TRH might control the hippocampal glutaminyl cyclase substrate and astrocyte reactivation [84]. AVP enhances cognitive and synapse functions in the hippocampus of APP/PS1 mice [85]. AVP expression in the neurons of the hypothalamus paraventricular nucleus could increase the release of adrenal stress hormones [86]. PMCH is linked to motivated behavior [87], social impairment, and the progression of psychiatric disorders, such as schizophrenia [88]. A previous study reported that the administration of insulin and insulin-like peptides led to an increase in the melanin concentration hormone (MCH) level [89]. Also, in the volcano plot, we observed several differentially expressed genes including *Dapk2* in the adipose tissue (Figure 1F). It was shown that DAPK2 is related to reductions in the adipocyte autophagic clearance system [90] and metabolic autophagy [91].

As shown in the volcano plot, the altered genes in the hippocampus of the IGF-1-injected APP/PS2 mice were roughly associated with synapse connectivity, the GABA system, astrocyte reactivation, neurogenesis, neural differentiation, the autophagy system, depressive behaviors, and cognition involving neuropsychiatric and neurodegenerative disease pathology. Also, the changed genes in the adipose tissue of the IGF-1-injected APP/PS2 mice were related to adipogenesis, mitochondrial dysfunction, and the autophagy system involved in the metabolic imbalance status.

In addition, we observed the ten most increased and decreased genes in the hippocampus (Figure 1G). The increased level of the *Trh* gene signifies a stable hippocampal GABA neurotransmitter system, improves memory function, and indicates a stable mood status in the IGF-1-injected APP/PS2 mice [83]. Also, the increased expression of the *Avp* gene signifies the enhancement of cognitive function and synaptic connectivity by the IGF-1 injections [85]. The increased expression of the *Kcnmb2* gene indicates the restoration of hippocampal dysfunction [92], and KCNMB2 plays a protective role in the brain aging response [93]. The increased expression of the *Folr1* gene indicates the increase in dopaminergic neurogenesis [94] from the IGF-1 injections. The increased expression of the *Ecel1* gene indicates the promotion of the axonal branching of neurons and brain synaptic connectivity [95].

The decreased expression of the *Pglyrp1* gene indicates a reduction in the immune response and inflammatory response in the hippocampus from the IGF-1 injections [96]. The reduced expression of the *Alg13* gene signifies that the epilepsy-related neuropsychiatric pathology was attenuated via the regulation of GABA receptors in the IGF-1-injected APP/PS2 mice [97].

We also observed the ten most increased and decreased genes in the adipose tissue (Figure 1G). The increase in the *Lcn2* gene indicates the activation of the thermogenic function and lipid metabolism in the adipose tissue by the IGF-1 injections [98,99].

Next, we performed a GO term analysis (Figure 2A,B). The top 15 enriched GO terms in the hippocampus were cellular response to growth factor stimulus, the regulation of cell differentiation, ErbB signaling, and the glial cell apoptotic process (Figure 2A). Previous studies have shown that IGF-1 boosts neuronal cell differentiation and neuronal stem cell proliferation [100,101]. Moreover, the ErbB signaling stimulated by IGF-1 is related to synaptic plasticity [102,103] and promotes long-term hippocampal depression and object recognition memory ability [104]. Additionally, IGF-1 might modulate glial apoptosis and mitochondrial function in astrocytes [105].

Several studies have shown that IGF-1 boosts hippocampal neurogenesis [106], restores spatial memory [48], and improves depressive-like behaviors through the regulation of serotonin [107]. Numerous studies have demonstrated that the administration of IGF-1 lessened brain atrophy [32], brain insulin resistance [108], apolipoprotein E-related neuropathology [109], and neurotransmitter secretion [110]. Other studies have mentioned that IGF-1 improved the long-term survival of hippocampal neurons [111] and spine maturation [112].

The highly enriched GO terms in the adipose tissue included the regulation of cell death, regulation of inflammatory response, and the cellular metabolic process (Figure 2A). One study mentioned that AD patients show less of the fat-browning process and adipose tissue atrophy compared with normal patients [25]. The fat browning-related genes in this study’s mRNA data indicate that IGF-1 promotes the fat-browning process of adipose tissue in the APP/PS2 mice compared with the control mice (Figure 2C). IGF-1 boosts the lipogenesis, adipocyte differentiation, and metabolic response in adipocytes [113]. Also, IGF-1 promotes thermogenesis and fat browning in adipose tissue and attenuates adipocyte cell death [114,115].

Moreover, we detected an increase in the protein level of p53 in the hippocampus and adipose tissue in IGF-1-injected APP/PS2 mice (Figure 2D). p53 regulates body weight, the fat-browning process [116], adipogenesis, and adipocyte differentiation and protects adipocytes against cell death due to stress conditions [117]. p53 can activate the IGF-1/AKT and mTOR signal pathways related to the autophagy system [118,119]. The p53-induced antioxidant pathway is impaired through the reduced expression of MnSOD in AD brains [120], and MnSOD is observed in the hippocampus of mild cognitive impairment due to low glutathione levels [121]. Considering our data and previous findings, the increased p53 protein level in the hippocampus of IGF-1-injected APP/PS2 mice might be related to the increased antioxidant response, mTOR autophagy system, and cell survival system from IGF-1 in the brain. Whereas the increased p53 protein levels in the adipose tissue of IGF-1-injected APP/PS2 mice may be associated with increased adipogenesis, fat browning, and the mTOR autophagy system and reduced adipocyte cell death.

Furthermore, we analyzed the lncRNAs and circRNAs that had a significantly altered expression in the hippocampus and adipose tissue of APP/PS2 mice treated with IGF-1 (Figure 4 and Figure 5). However, compared with the protein-coding genes, there were fewer lncRNAs and circRNAs whose expressions were significantly and commonly changed (*p* < 0.1) in both tissues. In particular, there were no circRNAs with commonly changed expressions. We identified four lncRNAs whose expressions were significantly and commonly changed; however, RP23-325D10.5 was changed in the opposite direction in the hippocampus and adipose tissue, suggesting that this lncRNA may regulate opposite functions in these tissues.

The lncRNA, RP23-177L19.1, also named Gm13375, resides at the opposite strand to the protein-coding gene Arhgap21 (Figure 4E). Interestingly, Arhgap21 has been suggested as a key regulator in various cellular pathways [122]. It was shown that ARHGAP21 had an inhibitory role during insulin secretion via glucose stimulation [123]. Since RP23-177L19.1 was also changed by the IGF-1 treatments in our study (Figure 4C) and other studies have shown that there is a regulatory relationship between protein-coding genes and lncRNA when they share the promoter sequence, it could be expected that there is also an association between Arhgap21 and this lncRNA. Another lncRNA, RP23-234K24.8 (2410006H16Rik), also known as Lrrc75-as1 (Figure 4E), was shown in our previous report to be involved in the regulation of vascular calcification and commonly changed in ischemic strokes and myocardial infarctions [124,125]. Thus, it is expected that this lncRNA may be an important regulator in various diseases and requires further study.

We also found that the inhibition of circKsr2 tended to reduce the protein levels of p53 and GFAP and increase the protein levels of SYP in the hippocampus of APP/PS2 mice (Figure 7). These results suggest that circKsr2 is involved in regulating p53 expression in the hippocampus. GFAP is increased in a damaged brain or tissue and is a reactive marker of astrogliosis [126]. The high expression of GFAP has been reported to be associated with the AD-related accumulation of Aβ [127]. The reduced GFAP protein levels in the hippocampus of circKsr2-inhibited APP/PS2 mice may be associated with the reduced accumulation of Aβ pathology. SYP is a marker of synaptic vesicles and plays an important role in brain learning and memory function [128,129]. SYP regulates neuronal differentiation, neuronal activity, and synaptic transmission, and a decrease in SYP is known to lead to impaired learning and memory abilities [129,130,131]. These results show that the inhibition of circKsr2 may be a factor that can regulate a cognitive dysfunction such as AD by changing the levels of proteins such as p53, GFAP, and SYP in the hippocampus of APP/PS2 mice.

Previous studies have demonstrated that chaperone proteins, including Hsp70 and YB-1, exhibit neuroprotective effects and therapeutic benefits in mouse models of Alzheimer’s disease (AD) [132,133]. It would be intriguing to investigate whether these chaperone proteins elicit variances in gene expression patterns across diverse tissues, as explored in our study. Further research is warranted to explore the potential of treating AD using hormones like IGF-1, alternative enzymes such as chaperones, and in conjunction with diverse therapeutic agents.

One limitation of our experiments pertains to the relatively small sample sizes of mice utilized. While several experiments demonstrated significant differences even with limited sample sizes, others revealed trends without reaching a high level of statistical significance. Addressing this aspect warrants attention in future studies to ensure the robustness and generalizability of findings.

## 4. Materials and Methods

### 4.1. APP/PS2 Mouse Care and IGF-1 Injections

Three-month-old male APP/PS2 mice, which overexpress a mutant form of the human Aβ precursor protein and human presenilin-2, were housed in the Laboratory Animal Research Center of Chonnam National University (CNU), under a 16 h light/8 h dark cycle at 23 °C with 60% humidity and given ad libitum access to food and water except when conducting behavioral experiments. We used only male mice in our study due to the anticipation that the outcomes might be susceptible to potential influence by the hormonal variability inherent in female mice, given the hormone-like nature of IGF-1. The APP/PS2 mice were administered with normal saline or IGF-1 (human recombinant, animal-free IGF-1, Cat. No. GF306, Sigma Aldrich, St. Louis, MO, USA). Subcutaneous injections of IGF-1 (at a dose of 50 μg/kg/day) were given once every week for two months as a suspension in a sterile saline solution [54]. We measured the blood glucose level and body weight of the APP/PS2 transgenic mice before and after IGF-1 treatments. In addition, mice were subjected to the marble burying test after eight weeks, and nest shredding behavior test, and then were sacrificed via cervical dislocation after isoflurane anesthesia.

### 4.2. Marble-Burying Behavior Test

The marble-burying behavior test was used to assess the obsessive–compulsive behavior of the animals. All mice were placed in a polypropylene cage (26 cm × 48 cm × 20 cm) with 20 glass marbles (15 mm DM, 5.2 g in weight) kept in 5 rows of 4 marbles evenly distributed on a 5 cm deep sawdust bed. The mice did not have access to food or drink. The mice were placed in the cage for 30 min. The test polypropylene cages were kept in a separate room from the housing area. The number of marbles buried at least 2/3 of the way within 30 min was used to measure the compulsive-like digging behavior score [67]. A score was given when the mice had buried the marbles a distance of 2/3 from the surface into the bedding material [134].

### 4.3. Nestlet-Shredding Behavior Test

A polycarbonate cage (26 cm × 48 cm × 20 cm) was filled with bedding material and a nestlet (cotton bed; 1 g) and had a filter-top cover placed on top of the cage. The mice were placed in the cage for 30 min. Water and food were withheld during the experiment. Scoring was measured by the percentage of the nestlet that was shredded, which was calculated by dividing the weight of the remaining unshredded nestlet by the starting nestlet weight [134]. The weight of the shredded material (g) was calculated in a previous study: the weight of the shredded material (g) = the initial weight of the material—the weight of the material taken after the treatment [67,134].

### 4.4. RNA Sequencing Analysis

The total RNA from the hippocampus of APP/PS2 mice was extracted using a TRIzol reagent (Thermo Fisher, Waltham, MA, USA), and its integrity was examined using an Agilent 2100 BioAnalyzer (Agilent, Santa Clara, CA, USA). The total RNA was applied to a Ribo-Zero Gold rRNA Removal Kit (Illumina, San Diego, CA, USA) to remove ribosomal RNA. The RNA sequencing libraries were prepared using a TruSeq Stranded Total RNA Kit (Illumina). The RNA libraries were paired-end sequenced with 100 sequencing cycles on a HiSeq 2500 system (Illumina).

### 4.5. Analysis of RNA Sequencing Data

Among the reads produced from the RNA sequencing, those with a low-quality score of FASTQ were eliminated using Trimmomatic (removed leading or trailing low-quality bases below quality 3, dropped read when the average quality per four bases of sliding window drops below 20, and dropped reads below the 36 bases long) [135]. The trimmed sequences were aligned to the mouse genome (mm10) via the spliced transcripts alignment to a reference (STAR) aligner [136]. Cuffnorm was used to estimate the normalized values of the fragments per kilobase of transcript per million mapped reads (FPKM) according to the GENCODE annotation (Release M17, GRCm38.p6) [137]. After obtaining FPKM values for each gene from the Cuffnorm algorithm, those transcripts with mean FPKM values of less than 1 or transcripts not detected in any samples were excluded from further analysis. The FPKM values of each sample in the control and IGF-1 treatment groups were used to determine the significance of the difference between the groups using a *t*-test in Microsoft Excel 2019.

### 4.6. Functional Analysis of Changed Genes

To select the genes with significant changes in expression in the APP/PS2 mouse model, the 499 genes (325 increased and 174 decreased genes) with significant changes in expression in the hippocampus and the 245 (141 increased and 104 decreased genes) genes with significant changes in expression in the adipose tissue were selected based on their *p*-values with a cut-off criterion of 0.05. Among the genes in the group with *p*-values less than 0.05, the top ten increased genes and the top ten decreased genes based on the value of their fold change were selected from the hippocampus and adipose samples. We performed a gene ontology (GO) analysis at the Molecular Signatures Database [138] using the 200 genes selected from both tissues in the order of the smallest *p*-value. Among the genes with a significantly changed expression in the hippocampus and adipose, a gene interaction network analysis was performed using the top ten genes that increased the most and the bottom ten genes that decreased the most. For this analysis, the GeneMANIA plugin in Cytoscape was used [55,56]. Those genes without interactions were omitted from the data presentation.

### 4.7. Functional Analysis of Changed Non-Coding RNAs

To select the protein-coding genes that correlated with RP24-325N9.5 expression, we calculated the Pearson correlation coefficient between RP24-325N9.5 and the expression level of each protein gene in all the samples from the hippocampus and adipose. For each of the top 100 genes with the highest positive correlation coefficient and the top 100 genes with the lowest negative correlation coefficient, a GO analysis was performed using MSigDB. To find the regulatory relationship between the circRNA and miRNA, we used the TargetScan algorithm [62]. After obtaining the sequence of each circRNA, we analyzed whether there was a site for miRNA seed binding in the sequence. From the analysis result, we selected only miRNAs with a 7mer-m8 site or an 8mer site in the circRNA sequence [62].

### 4.8. RT-PCR

The total RNA was isolated from mouse adipose tissue using a TRIzol reagent (Ambicon, Chislehurst, UK) according to the manufacturer’s instructions. The extracted RNA were transcribed to complementary DNA (cDNA) using random hexamer (Thermo Fisher Scientific) and RevertAid reverse transcriptase (Thermo Fisher Scientific). A real-time PCR was conducted to measure the gene expression using a Power SYBR green PCR master mix (Applied Biosystems, Waltham, MA, USA) and Step One Plus real-time PCR system (Applied Biosystems). The expression of *Ucp1* or *Prdm16* was normalized to the *Gapdh* expression levels. PCR primers were used as the following primers: Prdm16 (mouse), 5′-CGTCCATCGCGGAGAAATAC-3′ (Forward) and 5′-TTAGGCAGGAACTGGAAGGG-3′ (Reverse); Ucp1 (mouse), 5′-TGTTGTCTTCAGGGCTGAGT-3′ (Forward) and 5′-CTTCGGAAGTTGTCGGGTTC-3′ (Reverse); Gapdh (mouse), 5′-AATGTGTCCGTCGTGGATCT-3′ (Forward) and 5′-AGACAACCTGGTCCTCAGTG-3′ (Reverse). The semi-quantitative PCR was performed using Phusion High-Fidelity DNA Polymerase (Thermo Fisher Scientific) in the Mastercycler Nexus X2 (Effendorf). The PCR products were electrophoresed on 2% agarose gel and quantified using Image J software (V1.54d). The expression levels of circRNA and mRNA were normalized by that of *Gapdh*. The primer sequences are listed in Supplementary Table S1.

### 4.9. Western Blotting

The tissues were lysed in an ice-cold radioimmunoprecipitation assay buffer (Translab, Bengaluru, India) for ten minutes. Protein extracts were quantified using a bicinchoninic acid protein assay kit (Thermo Fisher Scientific). The protein (10–20 μg) was loaded on an 8–12% sodium dodecyl sulfate-polyacrylamide gel, which was then transferred onto a polyvinylidene difluoride (Merck Millipore, Burlington, MA, USA) membrane activated by methanol. The membranes were then blocked with 5% bovine serum albumin (GenDEPOT, Katy, TX, USA) for one hour at room temperature, followed by incubation with primary antibodies (1:1000) overnight at 4 °C. Primary antibodies against P53 (Santa Cruz, sc-126), glia fibrillary acidic protein (GFAP) (Santa Cruz, sc-33673), synaptophysin (SYP) (Millipore, MAB368), and glyceraldehyde 3-phosphate dehydrogenase (GAPDH) (Santa Cruz, sc-32233) were used. The membranes were incubated with a horseradish peroxidase-labeled secondary antibody (1:5000; Santa Cruz) for one hour at room temperature and were visualized using an ECL solution (Thermo Fisher Scientific) and Fusion Solo software 16.0.8.0 (Vilber, Marne-la-Vallée, France). Protein expression was normalized to GAPDH.

### 4.10. Brain Osmotic Pump Implantation

We prepared an osmotic pump (Alzet, Cupertino, CA, USA) containing the siRNA with a final concentration of 5 μM using the siRNA-siPORTNeoFX solution (Invitrogen, Waltham, MA, USA). The osmotic pump containing the siRNA and the brain infusion kit (Alzet) were combined to incubate in sterile saline overnight at 37 °C. The APP mice were anesthetized using 1.5–2% isoflurane anesthesia, and the brain infusion kit-osmotic pump assembly was inserted into the mice’s lateral ventricle (mediolateral 1.0 mm, anteroposterior 0.3 mm based on the bregma) using a stereotaxic instrument (Harvard Apparatus, Holliston, MA, USA).

### 4.11. Mouse Cytokine and Adipokine Array

The cytokine and adipokine array (R&D Systems, ARY006, and ARY013, Minneapolis, MN, USA) was performed according to the manufacturer’s instructions using APP/PS2 mice. Blood samples were clotted at room temperature for 15 min and then centrifuged at 10,000 rpm for 10 min to collect the plasma. The plasma was then diluted and incubated in a blocking solution containing the antibody detection cocktail for one hour at room temperature, followed by incubation with the cytokine or adipokine membrane overnight at 4 °C. After incubating the membranes in the streptavidin-horseradish peroxidase solution for 30 min at room temperature, the dot blots were visualized using an ECL solution and the Fusion Solo software 16.0.8.0.

### 4.12. Confirmation of the Circular Structure of circKsr2

The total RNA was mixed with RNase R (Biosearch Technologies, Hoddesdon, UK) and a 10× RNase R reaction buffer to digest linear RNAs. The control sample was mixed only with the buffer. The mixture was incubated at 37 °C for 10 min, and the RNase R was inactivated at 95 °C for 3 min. After the RNase R treatment, the expression of circKsr2 and *Ksr2* was determined via semi-quantitative PCR. The sequences near back-splicing junctions of circKsr2 were confirmed via the Sanger sequencing (Solgent, Daejeon, Republic of Korea) of the PCR product.

### 4.13. Cell Fractionation

To separate the Neuro-2A cells into the nucleus and cytoplasm, the cells were washed with PBS twice and collected in PBS. Cell pellets were treated with a cold lysis buffer [10 mM HEPES buffer (pH 7.9), 10 mM KCl, 1 mM DTT, and 0.1 mM EDTA] and incubated on ice for 25 min. Then, 10% Nonidet P-40 (NP-40) was added to the mixture and incubated for an additional 2 min. After centrifugation, the supernatant, which includes cytoplasmic RNAs, was treated with a TRIzol LS reagent (Invitrogen) for RNA isolation. The pellet was resuspended in K100 buffer D [20 mM Tris (pH 8.0), 100 mM KCl, and 0.2 mM EDTA] and then centrifuged to extract nuclear RNAs. Precursor *Gapdh* (Pre-*Gapdh*) was used as a control for the nuclear fraction, and *Gapdh* was used as a control for the cytoplasmic fraction.

### 4.14. siRNA Construction

siRNAs targeting the back-splicing junction sequences of circKsr2 were designed using the siDESIGN Center in Horizon Discovery (https://horizondiscovery.com/en/products/tools/siDESIGN-Center, accessed on 13 June 2023) and i-Score Designer (https://www.med.nagoya-u.ac.jp/neurogenetics/i_Score/i_score.html, accessed on 13 June 2023). If the hydrogen bonds at the 5’ end of the antisense strand were the same or more than those of the sense strand, one sequence at the 3’ end of the sense strand was modified to retain the antisense strand in the RNA-induced silencing complex (RISC). The designed siRNA and the AccuTarget negative control siRNA were synthesized by Bioneer. The sequences of siRNAs are listed in Supplementary Table S1.

### 4.15. Statistical Analysis

All data were analyzed using unpaired two-tailed *t*-tests with Welch’s correction between groups. Data were considered significant at * *p* < 0.05, ** *p* < 0.01, and *** *p* < 0.005 in the statistical analysis.

## 5. Conclusions

Taken together, we summarize that IGF-1 promotes adipogenesis and adipocyte cell survival and improves mitochondrial function, adipocyte differentiation, and the fat-browning process in AD adipose tissue. Additionally, IGF-1 tends to improve synaptic plasticity; lessen the inflammatory response and glial cell death; increase neuronal cell survival, neurogenesis, and neuronal differentiation; and activate the GABA system and mTOR autophagy signaling in AD hippocampi. Thus, we suppose that further studies on the roles of IGF-1 in the hippocampus and adipose tissue are needed to find novel therapeutic mechanisms for AD neuropathology.

## Data Availability

The data presented in this study are available within the article. The data that support the findings of this study are available from the corresponding author upon reasonable request.

## Supplementary Materials

The supporting information can be downloaded at: https://www.mdpi.com/article/10.3390/ijms25052567/s1.

## Abbreviations

Alzheimer’s disease (AD); Insulin-like growth factor-1 (IGF-1); Amyloid beta (Aβ); Central nervous system (CNS), Insulin-like growth factor-I receptor (IGF-1R); thyrotropin-releasing hormone (Trh); arginine vasopressin (Avp), pro-melanin-concentrating hormone (Pmch); valosin containing protein lysine methyltransferase (Vcpkmt); taperin (Tprn); CUB and zona pellucida like domains 1 (Cuzd1); prominin 1 (Prom1); placenta expressed transcript 1 (Plet1); potassium sodium-activated channel subfamily T member 2 (Kcnt2); death associated protein kinase 2 (Dapk2); PR/SET domain 16 (Prdm16); Uncoupling protein-1 (Ucp1).
