# Supplementary material for: Identification of IGF-1 Effects on White Adipose Tissue and Hippocampus in Alzheimer’s Disease Mice via Transcriptomic and Cellular Analysis
Source: Int J Mol Sci. 2024 Feb 22;25(5):2567. doi: 10.3390/ijms25052567 (PMC10931577; doi:10.3390/ijms25052567)
Supplement: Supplementary file 1 [file ijms-25-02567-s001.zip › Supplementary Figure S1.pdf]

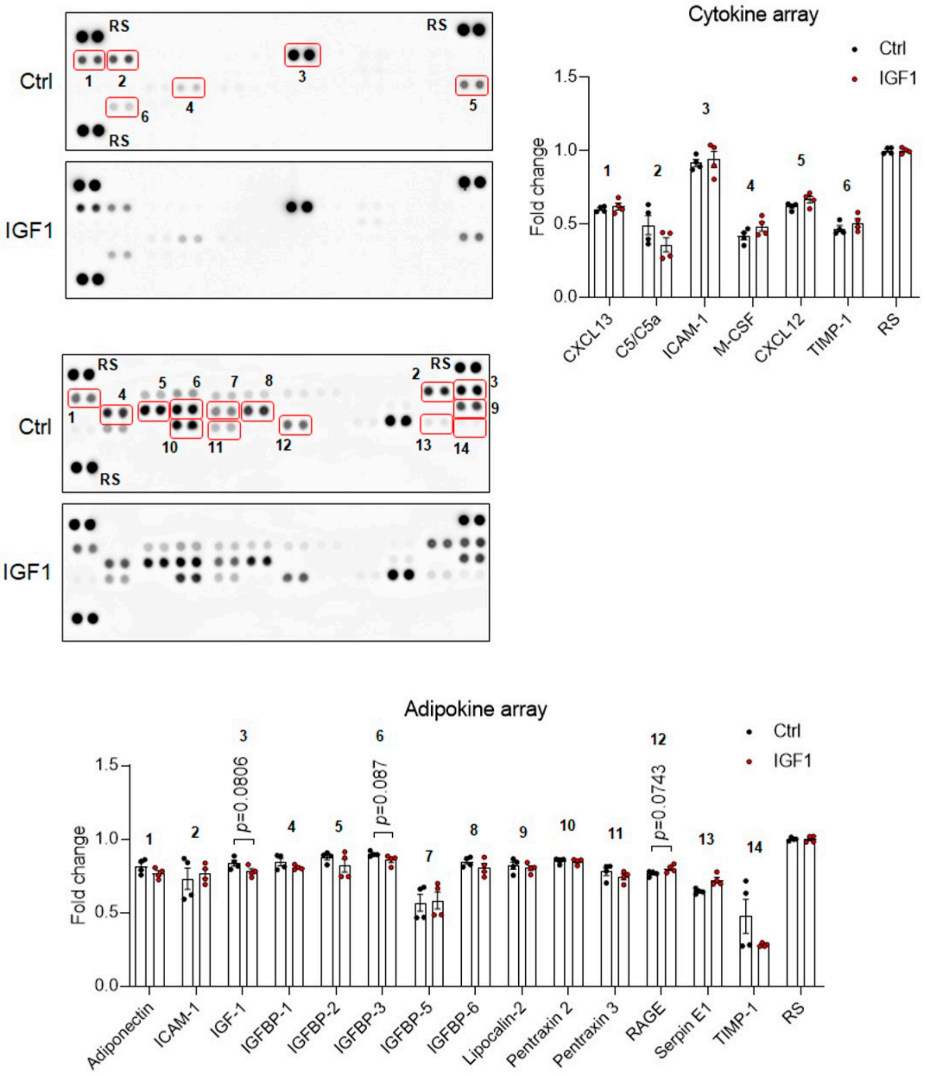

Supplementary Figure S1. The measurement of cytokines and adipokines in plasma of IGF-1-treated APP/PS2 mice. The level of C5/C5a was slightly decreased and the level of M-CSF and TIMP-1 was slightly increased in plasma of IGF-1-treated APP/PS2 mice. Among adipokines, the level of ICAM-1, RAGE and Serpin E1 were a little increased and the level of IGF-1, IGFBP-3 and TIMP-1 were slightly reduced in plasma of IGF-1-treated APP/PS2 mice.
