# Supplementary material for: Identification of IGF-1 Effects on White Adipose Tissue and Hippocampus in Alzheimer’s Disease Mice via Transcriptomic and Cellular Analysis
Source: Int J Mol Sci. 2024 Feb 22;25(5):2567. doi: 10.3390/ijms25052567 (PMC10931577; doi:10.3390/ijms25052567)
Supplement: Supplementary file 1 [file ijms-25-02567-s001.zip › Supplementary Figure S3.pdf]

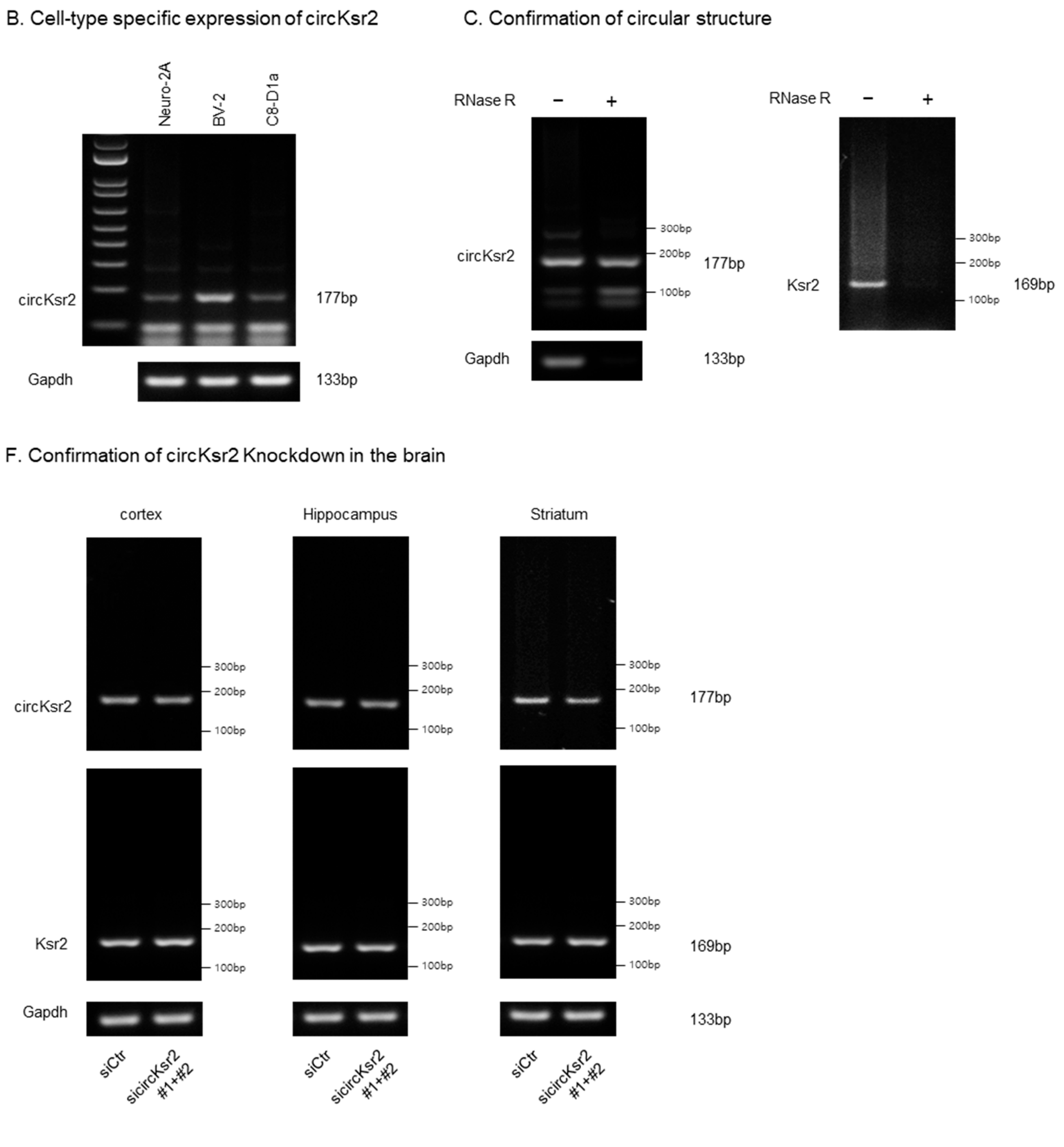

Supplementary Figure S3. Full-length blots of Figure 6.
