# Supplementary material for: Identification of IGF-1 Effects on White Adipose Tissue and Hippocampus in Alzheimer’s Disease Mice via Transcriptomic and Cellular Analysis
Source: Int J Mol Sci. 2024 Feb 22;25(5):2567. doi: 10.3390/ijms25052567 (PMC10931577; doi:10.3390/ijms25052567)
Supplement: Supplementary file 1 [file ijms-25-02567-s001.zip › Supplementary Table S1.pdf]

Supplementary Table S1. The list of primers and siRNAs used in this study.

**The list of siRNAs**		
Name	Forward sequence	Reverse sequence
Ksr2	GTGCCTTCCTGTAGTCTCTTG	AACAATGACCCCGTATGCTC
circKsr2	CCTTCTTCTTGCAGGACAGCTGT	CTTGGAGGACCTCCTGGAGATGA
Gapdh	AATGTGTCCGTCGTGGATCT	AGACAACCTGGTCCTCAGTG
pre-Gapdh	CCCTGGGATTAGGGTTGGAA	AAGGGCAAGGCTAAAGGTCA
**The list of primer sets**		
Name	Sense sequences	AntiSense sequences
sicircKsr2 #1	AAUCAGAGCAAGCUGGUGU=UU	UCACCAGCUUGCUCUGAUU=UU
sicircKsr2 #2	UGUGCACAAAUCAGAGCAA=UU	UUGCUCUGAUUUGUGCACA=UU
