# Supplementary figures and images for: Identification of IGF-1 Effects on White Adipose Tissue and Hippocampus in Alzheimer’s Disease Mice via Transcriptomic and Cellular Analysis
Source: Int J Mol Sci. 2024 Feb 22;25(5):2567. doi: 10.3390/ijms25052567 (PMC10931577; doi:10.3390/ijms25052567)

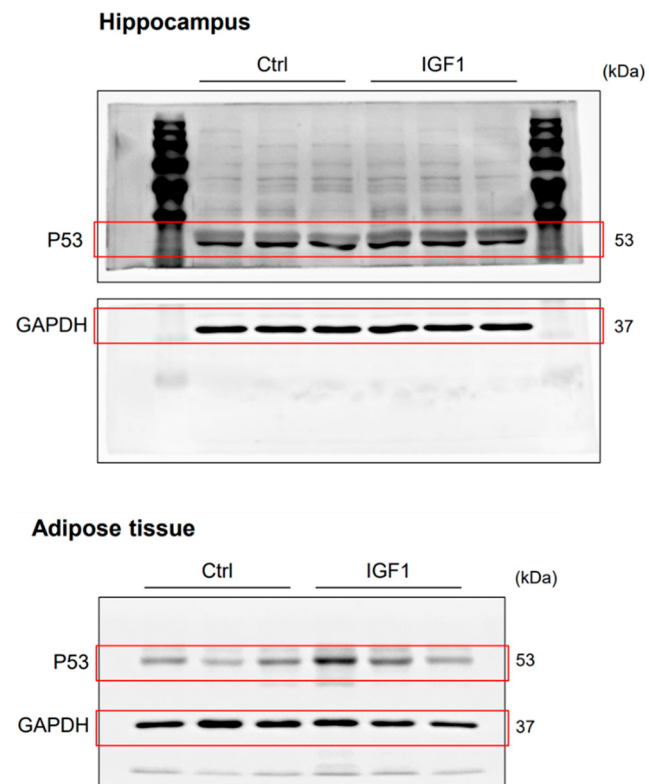

**Supplementary Figure S2.** Full-length blots of Figure 2D.

Supplement: Supplementary file 1 [file ijms-25-02567-s001.zip › Supplementary Figure S2.pdf]

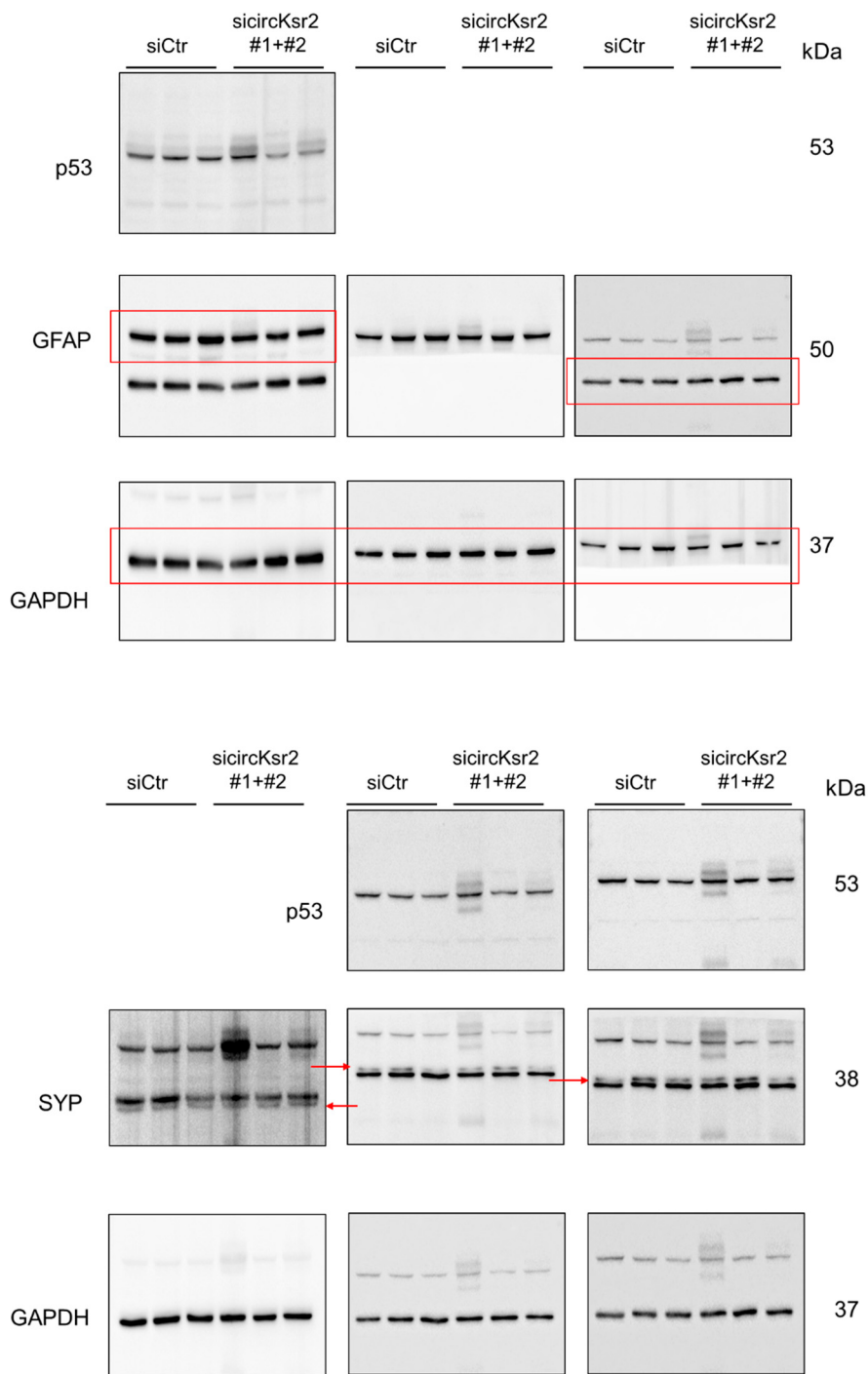

**Supplementary Figure S4.** Full-length blots of Figure 7.

Supplement: Supplementary file 1 [file ijms-25-02567-s001.zip › Supplementary Figure S4.pdf]

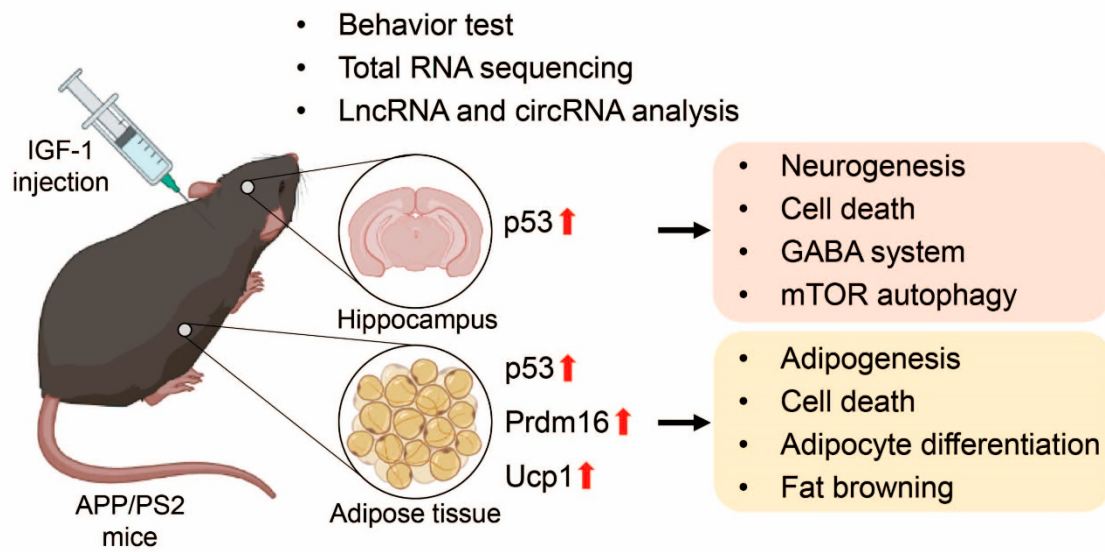

**Supplementary Figure S5.** Graphical summary of this study.

Supplement: Supplementary file 1 [file ijms-25-02567-s001.zip › Supplementary Figure S5.pdf]
